# In Vitro Comparison of Fluoride, Magnesium, and Calcium Phosphate Materials on Prevention of White Spot Lesions around Orthodontic Brackets

**DOI:** 10.1155/2020/1989817

**Published:** 2020-03-30

**Authors:** Selda Lale, Hikmet Solak, Evren Hınçal, Levent Vahdettin

**Affiliations:** ^1^Department of Orthodontics, Near East University Faculty of Dentistry, Nicosia, Northern Cyprus, Mersin 10, Turkey; ^2^Department of Restorative Dentistry, Eastern Mediterranean University Faculty of Dentistry, Famagusta, Northern Cyprus, Mersin 10, Turkey; ^3^Department of Mathematics, Near East University Faculty of Arts and Sciences, Nicosia, Northern Cyprus, Mersin 10, Turkey

## Abstract

One common negative side effect of orthodontic treatment with fixed appliances is the development of white spot lesions (WSLs) around brackets. This study is aimed at comparing the efficacy of various oral hygiene practices in preventing enamel demineralization around orthodontic brackets under similar in vitro conditions. The study included 90 extracted bovine incisors, which were randomized into six groups: fluoride toothpaste (FT), nonfluoride toothpaste (NFT), fluoride varnish plus fluoride toothpaste (FV+FT), CPP-ACP varnish plus fluoride toothpaste (CPP-ACP+FT), medical minerals gel plus nonfluoride toothpaste (MMG+NFT), and no intervention (control). All groups were subjected to demineralization and remineralization cycles. Visual appraisals were used to evaluate the changes in the enamel surface appearance at the beginning and end of the experiment. The changes in the demineralization degree were evaluated by measuring the Ca^+2^ concentration in the demineralization solution at different time points. The majority of teeth in the CPP-ACP+FT group exhibited no shift in appearance, whereas in the other groups, a slight change in enamel translucency was observed. At all the time points, the Ca^+2^ concentration in the demineralization solution in the CPP-ACP+FT group was the least among all other groups. At day 5, MMG+NFT's preventive efficacy was significantly higher than FV+FT's, but at days 10, 15, and 19, their efficacy was similar. However, at all the time points, MMG+NFT's efficacy was significantly higher than that of control, whereas FV+FT's efficacy was decreased at days 10, 15, and 19 and was close to the efficacy of control. To fight WSLs, early diagnosis was of great importance and examination of the tooth surface after air-drying for 5 s was recommended.

## 1. Introduction

One of the most common negative side effects of fixed orthodontic treatment is the development of incipient caries lesions [1]. Incipient caries lesions (white spots) are characterized by their opacity and mineral loss compared with healthy enamel. White spot lesions develop around orthodontic brackets, ligatures, bands, and other attachments because these appliances make it difficult to clean and potentiate bacterial biofilm accumulation on tooth surfaces [2].

Studies have shown that while it takes at least 6 months for caries to develop in a patient not submitted to orthodontic treatment, it takes around 1 month [2, 3] to develop in an orthodontic patient because of the difficulty these patients have with performing oral hygiene [4]. This information shows the rapid progression of this disease and the need for continual follow-up of these patients [2]. Investigations have put the incidence of white spot lesion (WSL) during orthodontic treatment with fixed appliances at 73% to 95% [5, 6].

Although there are many methods for the effective control of this initial stage of caries disease, prevention and early and accurate detection of WSLs is a great challenge to orthodontists [7].

There are many kinds of materials for preventing enamel demineralization at present, which can be categorized into 3 groups: (1) clinically applied agents, like fluoride varnish and fluoride foam. These agents negate the need for patient compliance and are quick and simple for dentists to apply. Bifluorid 12 (Voco, Cuxhaven, Germany) containing 6% sodium fluoride and MI Varnish (GC, Tokyo, Japan) containing 5% sodium fluoride are the most commonly used fluoride varnishes. (2) Self-applied agents under the guidance of dentists, e.g., medical minerals gel systems: these materials require close cooperation from the patients. R.O.C.S. Medical Minerals (“DRC-Group” company, Russia) contains bioavailable calcium, phosphate, and magnesium. (3) Daily oral hygiene practice agent, such as fluoride toothpaste, which normally contains 0.22% (1,000 ppm) and 0.312% (1,450 ppm) fluoride, or a nonfluoride toothpaste: Colgate Total (Colgate-Palmolive Company, NY, USA) contains 0.312% fluoride and R.O.C.S. Pro Brackets & Ortho (“DRC-Group” Company, Russia) contains calcium, phosphate, magnesium, and bromelain enzyme. Hence, routine oral hygiene practice, routine oral hygiene practice plus a professionally applied agent, and routine oral hygiene practice plus a self-applied agent under the guidance of dentists are 3 common ways to prevent WSLs.

The topical use of fluoride has become a widely recognized method for preventing enamel demineralization since the first clinical trial of fluoride varnish was published in 1964 by Schmidt [8]. The substitution of smaller spherical fluoride ions with hydroxide ions in hydroxyapatite strengthens the bonds within the lattice and reduces the solubility product. The formation of insoluble fluorapatite and calcium fluoride increases the enamel resistance for demineralization [9]. A significant prevention of the WSLs can be achieved during orthodontic therapy through fluoride varnish application [10–13]. A clinical study reported that when a high-dose fluoride varnish is placed around the orthodontic bracket at the beginning of treatment, the mean depth of demineralization was found to be approximately 40% less than that of the control group [14].

The casein phosphopeptide-amorphous calcium phosphate (CPP-ACP) system uses casein phosphopeptide (CPP), which is a milk derivative and bioactive agent to stabilize calcium and phosphate ions at high concentrations as amorphous calcium phosphate (ACP) in the oral environment. CPP is the carrier molecule for ACP. When the pH decreases, stabilized ions would be released; phosphorus ion buffers the pH condition, and calcium ion helps prevent demineralization and facilitate remineralization [15, 16]. Recently, more advanced fluoride varnishes with added CPP-ACP to NaF have been developed [17]. Their preventive and remineralization abilities have been proved by both in vitro and in vivo studies [18–20].

Besides these fluoride and CPP-ACP-based products, other components such as synthetic minerals composed of calcium, sodium, phosphorous, silica [21], and polymers [22, 23] have been investigated with respect to their ability to remineralize or reharden demineralized dental hard tissues. The assumed mode of action to prevent dental hard tissue loss is either to enhance the mineral uptake from the respective products (mainly CPP-ACP-containing products) or to increase the acid resistance of enamel (mainly fluoride and TiF4 or SnF2 containing products) to provide increased amounts of minerals, namely, calcium and phosphate, in the close surrounding or on top of the dental hard tissues for protection.

Based on the idea to provide an increased amount of minerals through the respective products, medical minerals gel is a newcomer in the field of prevention and treatment of caries and noncarious lesions by means of remineralization. The manufacturer claims that the mechanism of action of the system is based on the release of bioavailable calcium, phosphate, and magnesium and is indicated to treat early carious lesions. The remineralizing effect of the medical minerals gel was tested in a study by Kunin et al. [24]. It was observed that the medical minerals gel was effective in remineralizing the WSLs, improving teeth appearance. To our knowledge, no data from studies investigating the prevention effect of the medical mineral system are available.

However, when the WSLs are found during clinical practice, the lesions are found to have opacity, at least. At present, a few studies have focused on the degree of remineralization of the already-formed WSLs and the prognosis of remineralization therapy. Despite this, there are several questions as follows: (1) Can we prevent the WSLs before they occur? (2) Does prophylactic treatment during orthodontic treatment reduce the occurrence of the WSLs? (3) How should we choose the most effective and correct method when we have such a wide range of products and new materials?

Therefore, the aim of the present study was to investigate the preventive effect of the medical minerals gel and compare it with that of two types of varnishes (fluoride varnish and CPP-ACP varnish), which have been proved to be effective as protective agents, by using methods of observing the changes in visual score and amount of demineralization. If this study finds that medical minerals gel is effective in prevention, then this gel may possibly be added to the list of advised products that can be used to reduce demineralization and the occurrence of WSLs during orthodontic treatment.

## 2. Materials and Methods

### 2.1. Tooth Preparation

Ninety incisor teeth were collected from the mandibles of 3-year-old freshly slaughtered fifteen bovines (negative BSE test) and selected. The selection criteria included intact buccal enamel surfaces without the WSLs, visible cracks, or enamel irregularities.

The teeth were debrided of adherent bone and soft tissue with a no. 15 blade. The enamel surface of all teeth was then cleaned and polished using nonfluoridated pumice and a slow handpiece, rinsed with deionized water, and stored in 0.1% thymol solution at 4°C before use within 1 month.

**Figure 1:**
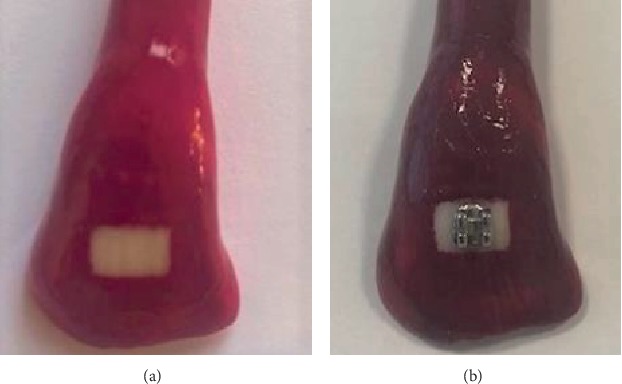
Images of teeth before and after bonding the brackets. (a) Before bonding the bracket: nail varnish painted around the exposed enamel surface. (b) After bonding the bracket: exposed enamel around the bracket represents the area of interest.

For standardization of the exposed enamel surface, a 4 × 6 mm^2^ window was created that was placed 5 mm gingivally to the incisal edge and in the mesiodistal center of the clinical crown. To avoid the interference of the general tissue demineralization of the crown, all teeth were painted with a thin coat of acid-resistant nail polish on all surfaces excluding the window area. Metal lower incisor brackets (3M Unitek, MN, USA) were bonded with a light-cured resin composite cement (Transbond XT, 3M Unitek, MN, USA) at the center of the exposed area [13] (Figure 1).

### 2.2. Visual Assessment

On the first day of the study, after the cleaning procedure, the exposed enamel surfaces of each tooth were examined visually with the aid of a light, air-drying and dental probe. The visual assessment was conducted by two examiners (one of whom was “blinded”) in accordance with the scoring criteria established by Ekstrand et al. [25] (listed in Table 1). Only lesions scoring 1 were included in the study. The same visual assessment was conducted at day 19 to evaluate the color and opacity changes of enamel around brackets.

### 2.3. pH Cycling Model

In the oral environment, after consumption of food, oral bacteria decompose sugar and produce acid, causing pH to decline and demineralization occurs further. However, there is also subsequent remineralization during long periods of exposure to saliva during the rest of the day. In order to imitate the oral environment in vitro and carry out remineralization process in artificial saliva (AS), all the teeth were immersed in the demineralization solution (DS) for 8 h and in AS for nearly 15 h every day. This procedure is called pH cycling [12].

### 2.4. Demineralization Cycle

After the materials were applied as described later, the groups were cycled in a 25 ml DS at 37°C for 8 h per day; each tooth was placed in a separate beaker. The DS was based on lactic acid and adjusted to pH 4.5 with sodium hydroxide [12]. The solution was refreshed daily during the experimental period of 19 d.

### 2.5. Remineralization Cycle (Artificial Saliva)

Following 8 h of exposure to the demineralization cycle, the teeth in each group were rinsed with deionized water and placed in AS in separate beakers that consisted of 20 mmol/l NaHCO3, 3 mmol/l NaH2PO4, and 1 mmol/l CaCl2 at neutral pH [13] for approximately 15 h until the next demineralization cycle [12].

### 2.6. Assigning to Groups of Treatments and Cycling Procedure

Under the same severity, six groups were established based on the prevention method:

1. No intervention (referred to as Control)
2. Fluoride toothpaste (Colgate Total, Colgate, USA) group (referred to as FT)
3. Nonfluoride toothpaste (R.O.C.S. Pro Brackets & Ortho, DRC-Group, Russia) group (referred to as NFT)
4. Fluoride varnish (Bifluorid 12, Voco, Germany) plus F toothpaste (referred to as FV+FT)
5. CPP-ACP varnish (MI Varnish, GC, Japan) plus F toothpaste (referred to as CPP-ACP+FT)
6. Medical minerals gel (R.O.C.S. Medical Minerals, DRC-Group, Russıa) plus non-F toothpaste (referred to as MMG+NFT)

Each group contained 15 teeth. The duration of the experiment was 19 d. The materials (as listed in Table 2) were applied to the exposed enamel surface around each bracket. All the groups were cycled daily as described before. Varnishes were applied once, at the beginning of the experiment. Medical minerals gel was applied daily according to the manufacturer's instructions and 30 min before starting the next demineralization cycle. Both toothpastes were applied daily, during the remineralization cycle, after immersing in AS for 30 min.

The details of the processes performed for each group during the study are as follows:

For the control group, all the teeth were subjected to demineralization and remineralization cycle daily and received no treatment during experiment period.

For the FT group, after 30 min of immersing the teeth in AS solutions, the teeth were individually removed from the beakers, brushed with a soft-bristled toothbrush and a pea-sized amount of F toothpaste for 2 sec per tooth, rinsed with deionized water for 5 sec, then immersed again into the AS solutions to complete approximately 15 h of the remineralization cycle [12].

For the NFT group, the teeth were brushed with the nonfluoride toothpaste using the same brushing method, as described for group 2.

For the FV+FT group, the fluoride varnish (Bifluorid 12) was applied to the exposed enamel around the brackets with a thin layer and distributed homogeneously with an applicator brush. This varnish was allowed to dry for 15 min as directed by the manufacturer. During the experiment, each tooth was brushed daily with the fluoride toothpaste, as described for group 2.

For the CPP-ACP+FT group, the CPP-ACP varnish (MI Varnish) was applied in the same manner as described for group 4. During the experiment, each specimen was brushed with the fluoride toothpaste, as described for group 2.

For the MMG+NFT group, the teeth were dried, and a pea-sized amount of R.O.C.S. Medical Minerals gel was applied around the brackets by a cotton swab, left undisturbed for 30 min on the tooth surfaces, and then rinsed with deionized water for 5 sec. This procedure was applied daily according to the manufacturer's instructions, 30 min before the next demineralization cycle, as mentioned previously. Each tooth was brushed with the nonfluoride toothpaste using the same method as described for group 2.

### 2.7. Determination of Calcium Loss (Demineralization)

To assess the amount of demineralization, a colorimetric technique was used [26, 27]. The procedures were carried out at the Medical Biochemistry Laboratory at Near East University Hospital. After 8 h of exposure to the demineralization cycle, the teeth were removed from each beaker. Calcium content of each beaker was measured with the Abbott Architect c8000 biochemistry autoanalyzer system using the Arsenazo III method [28].

**Figure 2:**
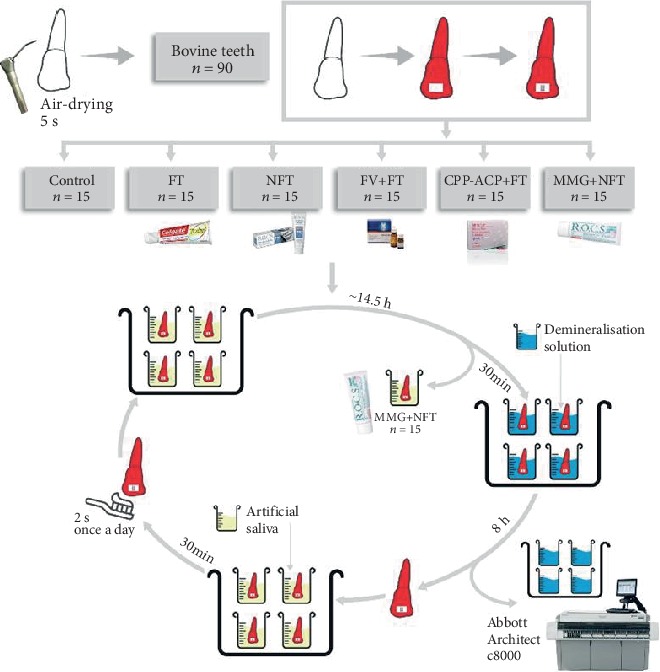
The experimental procedure of this study.

The Ca^+2^ content of DS samples was analyzed for each group at days 5, 10, 15, and 19 to observe change of demineralization according to the days. The lack of Ca^+2^ in DS is estimated as a measure of the demineralization inhibitory effect of the materials [29]. The experimental procedures were shown in Figure 2.

### 2.8. Statistical Evaluations

Descriptive statistics were calculated. Median differences among the pre- and posttreatment visual assessment data for each of the groups were compared using the Bonferroni-adjusted Wilcoxon Signed-Rank test (*p* < 0.001).

The ANOVA test with the post hoc Bonferroni test revealed the significant differences between groups for average demineralization. The results were considered significant at *p* = 0.05. Power analysis was conducted on the Wilcoxon test data to ensure that the sample size and the magnitude of the observed effect were sufficient. The statistical analysis was performed using SPSS software (version 24.0.1, SPSS, Chicago, Ill).

## 3. Results

### 3.1. Visual Assessment

**Figure 3:**
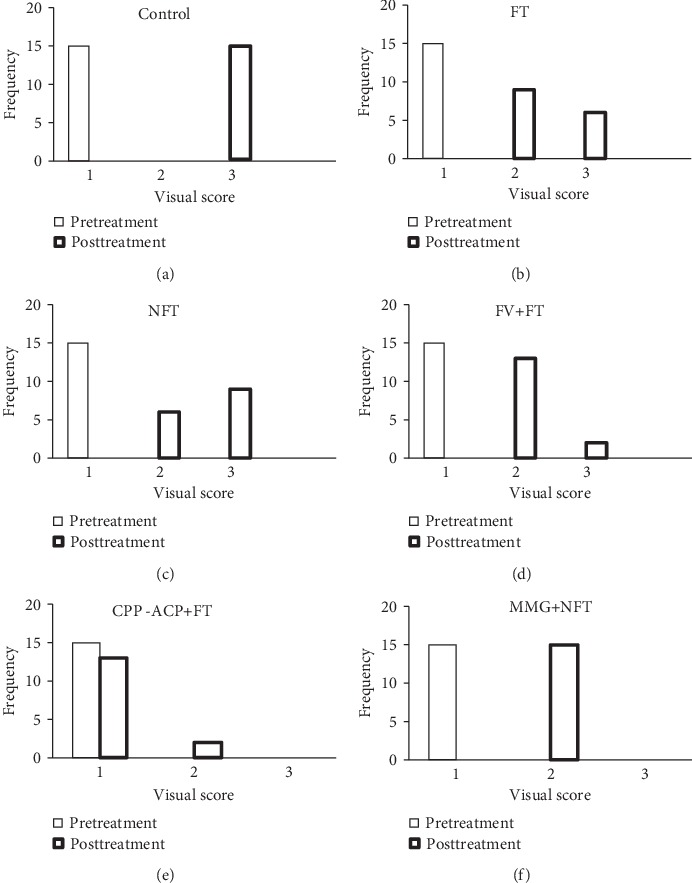
Frequency distribution plots for the pre- and posttreatment visual scores for (a) control group, (b) FT group, (c) NFT group, (d) FV+FT group, (e) CPP-ACP+FT group, and (f) MMG+NFT group.

Frequency plots showing the distributions of pre- and posttreatment visual assessment scores for each treatment regime are given in Figure 3. A summary of the results of the corresponding statistical analysis via the Bonferroni-adjusted Wilcoxon Signed-Rank test is listed in Table 3.

These results indicate that the majority of teeth in the CPP-ACP+FT group exhibited no shift in appearance, whereas in the other groups (control, FT, NFT, FV+FT, and MMG+NFT), the visual appearance of the enamel translucency was significantly changed (*p* < 0.001).

### 3.2. Ca Loss Measurement (Demineralization)

**Figure 4:**
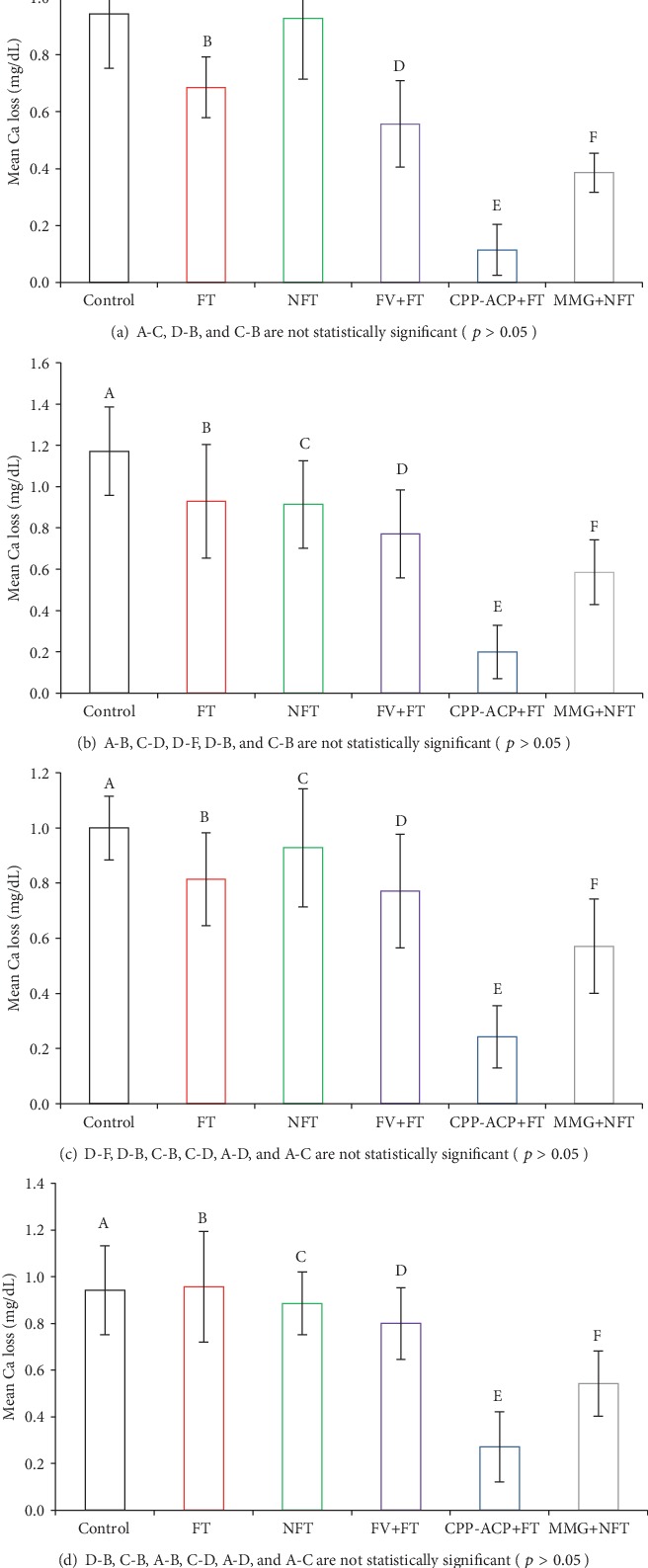
Ca loss measurement (mean ± sd; *n* = 15) plots of treatment groups for (a) day 5, (b) day 10, (c) day 15, and (d) day 19. Different letters are used in pairwise comparisons of groups.

The degrees of demineralization that were evaluated by measuring the Ca^+2^ concentration in the demineralization solution at days 5, 10, 15, and 19 for each group and a summary of the corresponding post hoc Bonferroni test results are shown in Figure 4.

The results of the Ca^+2^ loss measurements confirm the findings of the visual appraisal and indicate that the extent of prevention afforded by the six different treatment regimens investigated in this study for all time points of measurement is in the following order: control⟶NFT⟶FT⟶FV + FT⟶MMG + NFT < CPP − ACP + FT.

At all the four time points of measurement (days 5, 10, 15, and 19), the significantly lowest Ca^+2^ concentration in the demineralization solution was observed for the CPP-ACP+FT group.

No advantage was observed for the use of the FV as a supplement to the daily application of the FT during experiment period, but when the MMG was used as a supplement to daily application of the NFT, additional protection was observed at all the time points of measurement.

At day 5, MMG+NFT's preventive efficacy was significantly higher than FV+FT's, but at days 10, 15, and 19, their efficacy was similar. However, at all the time points, MMG+NFT's efficacy was significantly higher than that of control, whereas FV+FT's efficacy was decreased at days 10, 15, and 19 and was close to the efficacy of control.

## 4. Discussion

In the experimental caries studies, besides the use of extracted premolar teeth, bovine incisors were also used [13, 30] such as the present one. Although there are some differences between the bovine and human enamel, Featherstone and Mellberg [31] reported that bovine teeth were suitable for comparative purposes in demineralization studies. In addition, we have benefited from the advantages of using bovine incisors. As being larger than premolar teeth, it provides a wider surface for experimental work and is easy to obtain and there is no history of caries or fluorosis that can affect their chemical properties.

This study compared the efficacy of various oral hygiene practices in the prevention of orthodontic incipient carious lesions (WSLs). Both qualitative and quantitative results support each other.

A standard oral hygiene program was devised for the FT and NFT groups in this study. During the experiments, although numerical values showed less mineral loss in both the toothpaste groups than the control group, the statistical evaluation results suggested that the AS and both the toothpastes (fluoride and nonfluoride) were inefficient for the prevention of demineralization. Brushing and masticatory movement caused the abrasion of the tooth surface, resulting in the loss of demineralized enamel structure [32, 33]. The complex chemistry of the demineralization and remineralization processes of dental enamel and the specific mechanisms by which fluoride species operate in these processes are not yet fully understood; however, the role of extraneous fluoride ions in the prevention of early caries is universally accepted [14, 34–37]. When the concentration of fluoride ions is relatively high in preventive materials, they combine with calcium ions to form calcium fluoride, promoting remineralization. Nonfluoride toothpastes claim to be protective and have a remineralizing effect but that depend solely on their ability to provide calcium, phosphate, and magnesium ions to fill up defects in the enamel crystals. However, when the fluoride content or mineral-enriched content in pea-sized fluoride or nonfluoride toothpaste used for brushing teeth was about 2–3 mg, gargling after brushing further reduced the content that could bind to the enamel surface. Therefore, the therapeutic effect was not significant. Previous studies found that brushing with toothpaste alone cannot prevent the progression of the WSLs found during orthodontic treatment [38–40]. Hence, added preventive treatments are needed to prevent WSLs.

Fluoride varnish provides a temporary reservoir of highly concentrated fluoride ions in direct contact with the enamel surface, and these fluoride ions can diffuse into the hydroxyapatite crystals. The substitution of free fluoride ions with hydroxide ions decreases the crystal volume, increases the stability, and reduces the solubility of the apatitic crystals [41]. The clinical application of the fluoride varnish as an adjunct to good oral hygiene has been reported to provide an advantage in both the prevention and reversal of WSLs [14, 36, 42]. However, this finding is not unanimous [43]. One concern regarding fluoride therapy for the treatment of WSLs is the potential hypermineralization of the surface layer in the presence of high concentrations of fluoride ions, which physically blocks the subsequent ingress of calcium and phosphate ions into the body of the lesion [44]. Accordingly, some researchers have conjectured that high doses are recommended to inhibit initial lesion formation, although optimum fluoride doses and delivery mechanisms have yet to be established [44, 45].

The findings of this study have indicated that one-time application of 6% sodium fluoride varnish during the 19 experimental days as a supplement to the fluoride toothpaste did not provide any preventive advantage. Neither the visual appearance nor the Ca loss measurement of the teeth in the fluoride varnish plus F toothpaste group differed significantly from those of the F toothpaste group. The results of this study are in agreement with those of the study carried out by Huang et al. [43], who found that a single application of 5% sodium fluoride varnish in an 8-week follow-up had no impact on postorthodontic WSL regression. Conversely, other studies have indicated that fluoride varnishes can have a beneficial impact on lesion regression during and following orthodontic treatment [14, 46]. Supplementary fluoride varnish treatment is clearly an advantage for noncompliant patients with early carious lesions; however, it may be unnecessary to those who are observing stringent oral hygiene regimens, which include fluoridated dentifrices.

According to the results of this study, the combined use of fluoride varnish plus F toothpaste and the combined use of MMG plus non-F toothpaste showed a statistically similar preventive effect after day 5. However, numeric results show that medical minerals gel plus NFT exhibited more protection than fluoride varnish plus FT. The fluoride varnish formed a firm thin layer of varnish, which was stuck to the enamel surface after application, but because of the movement of buccal muscle and the tongue, mastication, saliva wash, and oral hygiene practice, the fluoride varnish is likely to be removed in a short period of time due to the complicated oral environment and movement. It has been reported that the fluoride varnish only remains in situ for up to 24 h [47]. In this experiment, the mechanical friction caused by tooth brushing leads to the gradual stripping of the Bifluorid 12 film. After 1 week, the varnish coat was unevenly exfoliated; after 1 month, it was completely removed. According to the clinical research [46, 48], the usage frequency of the fluoride varnish for preventing or treating WSLs is every 6 months. The fluoride ion could not be recharged on time, which led to the weakening of the remineralizing effect. However, by applying the medical minerals gel, the demineralized area was able to receive large amounts of calcium, phosphate, and magnesium ions regularly; hence, the protection was continuous. Hence, the varnish efficacy decreased compared with the medical minerals gel group after day 5.

The application of the CPP-ACP varnish plus F toothpaste yielded significantly less demineralization and showed no change in visual assessment compared with the fluoride varnish plus F toothpaste. Because the two varnishes presented approximately the same fluoride concentration and were applied with the same frequency, the difference in their composition could be a probable explanation for their different efficiency. It can be concluded that the CPP-ACP greatly increased the anticaries properties of the product and considerably diminished the mineral loss. The results of this study are consistent with those of the study carried out by Pithon et al. [30], who reported that a single application of the CPP-ACP fluoride varnish was more effective than Duraphat (5% sodium fluoride varnish without CPP-ACP) in reducing the depth of caries lesions around orthodontic brackets. Wierichs et al. [11], in an in vitro study, compared the caries-preventive effect of different varnishes on artificial dentin caries and found that CPP-ACP varnish could remineralize artificial dentin caries-like lesions under net-demineralizing conditions, thereby indicating that the CPP-ACP and SDF varnishes might be protective for high-caries-risk patients. However, our results are in contrast with those of a previous study [49] using initially demineralized human enamel. In that study, significantly less mineral gain was observed with CPP-ACP compared to that with NaF. The mentioned study used the longest remineralizing period of 22 h of remineralization in 1 day. The design of a pH cycling model has a major impact on the response, and the model likely had a strong remineralizing environment. The results of the previous studies therefore indicate that the more demineralizing the pH cycling conditions, the higher the demineralization inhibitory (remineralization-enhancing) effect of CPP-ACP seems to be, especially when compared to NaF. Therefore, it can be speculated that varnishes containing CPP-ACP are reservoirs of bioavailable Ca^+2^ and PO^−4^, especially when demineralization predominates.

These results suggest the importance of detecting WSLs at an early stage. Since visual examination is the most common detection method, orthodontists should carefully examine the tooth surface after air-drying for 5 sec. The gingival side of the bracket needs to be paid extra attention by both orthodontists during examination and patients during oral hygiene practice.

## 5. Conclusions

The findings of this 19-day in vitro study have indicated that

1. One-time application of the CPP-ACP varnish as an adjunct to the fluoridated dentifrice is significantly the most protective treatment against demineralization
2. Daily application of medical minerals gel plus non-F toothpaste exhibited more protection than fluoride varnish plus F toothpaste
3. There was no clinical advantage of one-time application of 5% sodium fluoride varnish as a supplement to daily application of fluoride toothpaste
4. Both toothpastes (fluoride and nonfluoride) had a weak preventive effect

This study provides a guideline to prevent the highly prevalent white spot lesions by effectively combining different methods. In addition, it provides vital knowledge about medical minerals gel, which is a relatively new product in the dental care field. The results of this study may be helpful to design new materials, which combine the beneficial properties of different existing materials, for prevention of the white spot lesions.

## Figures and Tables

**Table 1:** Visual examination criteria used in the selection and assessment of teeth [25].

Score	Visual assessment criterion
1	No, or slight, change in enamel translucency after air-drying for 5 seconds
2	Opacity or discoloration hardly visible on the wet surface but visible after air-drying
3	Visible opacity or discoloration without air-drying
4	Localised enamel breakdown with opacity or greyish discoloration from the underlying dentin
5	Cavitation in opaque or discolored enamel exposing the dentin

**Table 2:** Composition of commercially available products used in this study.

Material	Composition
Fluoride toothpaste (Colgate Total, Colgate-Palmolive Company, NY, USA)	Sodium fluoride (1450 ppmF−), water, glycerin, hydrated silica, sorbitol, PVM/MA copolymer, sodium lauryl sulfate, aroma, carrageenan, sodium hydroxide, propylene glycol, cellulose gum, triclosan, sodium saccharin, limonene, and CI 77891 (white pigment)
Nonfluoride toothpaste (R.O.C.S. Pro Brackets & Ortho, “DRC-Group” company, Russia)	Sorbitol, silica, glycerin, aqua, xylitol, cocamidopropyl betaine, aroma, xanthan gum, calcium glycerophosphate, bromelain, magnesium chloride, sodium saccharin, sodium benzoate, O-cymen-5-ol, and titanium dioxide
Fluoride varnish (Bifluorid 12, Voco, Cuxhaven, Germany)	Sodium fluoride and calcium fluoride (6%), ethylacetate, pyroxylin, fumed silica, clove oil, and isoamylpropionate
Casein phosphopeptide-amorphous calcium phosphate(CPP-ACP) varnish (MI varnish, GC, Tokyo, Japan)	Sodium fluoride and CPP-ACP (5%), polyvinyl acetate, hydrogenated rosin, ethanol, and silicon dioxide
Medical minerals gel (R.O.C.S. Medical Minerals; “DRC-Group” company, Russia)	Aqua, glycerin, xylitol, hydroxyethylcellulose, calcium glycerophosphate, polysorbate-20, aroma, methylparaben, magnesium chloride, and hydroxypropyl guar

**Table 3:** Median pre- and posttreatment visual examination scores in each group (used products), interquartile ranges, and significance levels.

Treatment regime	Control	FT	NFT	FV+FT	CPP-ACP+FT	MMG+NFT
Pretreatment median (interquartile range)	1 (1-2)	1 (1-2)	1 (1-2)	1 (1-2)	1 (1-2)	1 (1-2)
Posttreatment median (interquartile range)	3 (2-3)	2 (2-3)	2 (2-3)	2 (2-3)	1 (1-2)	2 (1-2)
Sample size (*n*)	15	15	15	15	15	15
Significance level (*p*)	<0.001	<0.001	<0.001	<0.001	0.157	<0.001

## Data Availability

The data used to support the findings of this study are available from the corresponding author is upon request.
